# Intraamygdaloid Oxytocin Increases Time Spent on Social Interaction in Valproate-Induced Autism Animal Model

**DOI:** 10.3390/biomedicines11071802

**Published:** 2023-06-23

**Authors:** Dávid Vörös, Orsolya Kiss, Tamás Ollmann, Kitti Mintál, László Péczely, Olga Zagoracz, Erika Kertes, Veronika Kállai, Bettina Réka László, Beáta Berta, Attila Toth, László Lénárd, Kristóf László

**Affiliations:** 1 Medical School, Institute of Physiology, University of Pécs, Szigeti Str. 12, 7602 Pécs, Hungary; voros.david@pte.hu (D.V.); kiss.orsolya@pte.hu (O.K.); tamas.ollmann@aok.pte.hu (T.O.); kitti.mintal@aok.pte.hu (K.M.); laszlo.peczely@aok.pte.hu (L.P.); olga.zagoracz@aok.pte.hu (O.Z.); erika.kertes@aok.pte.hu (E.K.); veronika.kallai@aok.pte.hu (V.K.); bettina.csetenyi@aok.pte.hu (B.R.L.); beata.berta@aok.pte.hu (B.B.); attila.toth@aok.pte.hu (A.T.); laszlo.lenard@aok.pte.hu (L.L.); 2 Neuropeptides, Cognition, Animal Models of Neuropsychiatric Disorders Research Group, Medical School, Institute of Physiology, University of Pécs, 7602 Pécs, Hungary; 3 Neuroscience Center, University of Pécs, 7602 Pécs, Hungary; 4 Learning in Biological and Artificial Systems Research Group, Medical School, Institute of Physiology, University of Pécs, 7602 Pécs, Hungary; 5 Cellular Bioimpedance Research Group, Szentágothai Research Center, University of Pécs, 7602 Pécs, Hungary; 6 Molecular Endocrinology and Neurophysiology Research Group, Szentágothai Center, University of Pécs, 7602 Pécs, Hungary

**Keywords:** oxytocin, autism, amygdala, social interaction, valproate, rat model

## Abstract

Autism spectrum disorder (ASD) is a pervasive neurodevelopmental disorder that affects about 1.5% of children worldwide. One of the core symptoms is impaired social interaction. Since proper treatment has not been found yet, an investigation of the exact pathophysiology of autism is essential. The valproate (VPA)-induced rat model can be an appropriate way to study autism. Oxytocin (OT) may amend some symptoms of ASD since it plays a key role in developing social relationships. In the present study, we investigated the effect of the intraamygdaloid OT on sham and intrauterine VPA-treated rats’ social interaction using Crawley’s social interaction test. Bilateral guide cannulae were implanted above the central nucleus of the amygdala (CeA), and intraamygdaloid microinjections were carried out before the test. Our results show that male Wistar rats prenatally exposed to VPA spent significantly less time on social interaction. Bilateral OT microinjection increased the time spent in the social zone; it also reached the level of sham-control animals. OT receptor antagonist blocked this effect of the OT but in itself did not significantly influence the behavior of the rats. Based on our results, we can establish that intraamygdaloid OT has significantly increased time spent on social interaction in the VPA-induced autism model, and its effect is receptor-specific.

## 1. Introduction

According to the fifth edition of the Diagnostic and Statistical Manual of Mental Disorders (DSM-5), autism spectrum disorder (ASD) is characterized by the autistic dyad, which consists of impairment of social interaction and communication and the presence of repetitive patterns in behavior, interest, or activities [1]. ASD is a pervasive disorder; therefore, it affects the whole personality of the patients and widely influences their lifestyle. Since no specific biological marker is known, the diagnosis is based on behavioral symptoms [2,3]. Being a spectrum disorder, the severity of symptoms is variable, but in most cases, impairment of social interaction is present [3]. ASD can also co-occur with many conditions, including fragile X syndrome, anxiety, schizophrenia, sleep disturbances, epilepsy, and mental retardation [4]. The economic cost of ASD is rather high (special education, healthcare, and community services) [5]. Proper medical treatment is still not solved; therefore, a particular understanding of pathomechanism would be necessary. The incidence of ASD is about 1.5% among children, and it tends to rise continually [6]. Boys have a fourfold higher risk of developing ASD than girls [6]. Since it is a neurodevelopmental disorder, the first symptoms appear in early childhood [6]. In the development of ASD, many risk factors can take place, such as older parental age, low birth weight, genetics, environmental pollution, and fetal exposure to infections and drugs (e.g., valproate) [7,8]. Valproate (VPA) is a broad-spectrum antiepileptic drug that is also used in the therapy of bipolar disorder, migraine, dementia, or neuropathic pain [9]. Studies have shown that it has a high teratogenic risk, and it can significantly enhance the development of ASD [10,11]. Based on the above-mentioned results, VPA seemed to be an effective drug to induce autism in rodents in order to study the pathophysiology of ASD. Kim et al., have shown that the most significant autism-like symptoms without toxicological side effects appear if the dams receive 500 mg/bwkg intraperitoneal (ip.) VPA on the 12.5th day of gestation [12]. Due to the intrauterine VPA exposure (500 mg/bwkg ip., gestational day 12.5), rats could show the following core signs of ASD: repetitive behavior; anxiety; decreased sociability; and impaired communication [13]. Social interaction and communication deficits could appear in various forms. Several studies have shown profoundly impaired social play in the VPA rat model that would be crucial for proper development [14,15]. Hirsch et al., have found impaired social transmission of food preference in intrauterine VPA-treated rats [16]. Impairment of communication has been studied by measuring ultrasonic vocalizations emitted by animals. Studies have shown that mice and rats prenatally exposed to VPA made fewer calls when separated from the dams and siblings [17,18,19].

Our examined brain structure was the amygdala which is located in the medial temporal lobe and is part of the limbic system [20]. We specifically examined the central nucleus of the amygdala (CeA), which has connections with the other nuclei of the amygdala, the hypothalamus, and vegetative centers in the brain stem; therefore, it plays an essential role in the assessment of information [21]. It is also closely linked to social interaction, which is impaired in ASD [22]. Post-mortem studies have found a decreased number of neurons in the amygdala, cerebellum, and frontal lobe in patients with ASD [23,24]. Furthermore, it plays a role in establishing and maintaining eye contact and in anxiety as well, both of which are associated with ASD [25]. Amygdala is rich in oxytocin receptors (OTR); therefore, not surprisingly, it plays an essential role in processes ruled by the oxytocinergic system as well [26,27,28].

Oxytocin (OT) is a nonapeptide mostly produced by the paraventricular, supraoptic, and accessory nuclei of the hypothalamus [29]. Besides its well-known peripheral effects, OT plays an important role in social recognition, parental behavior, and social interaction, among others [28,30]. The central oxytocinergic system consists of projections from the hypothalamus to the hippocampus, the ventral tegmental area, the nucleus accumbens, the prefrontal cortex, and the amygdala [24,31,32,33]. The impairment of the oxytocinergic system can be associated with such psychiatric disorders as ASD, addiction, depression, and anxiety [34,35]. OT develops its effects mainly via its own receptor (OTR), which is part of the seven-transmembrane receptor superfamily [29]. Although several preclinical and clinical studies have investigated the effect of intranasal OT therapy [36,37,38], results are inconsistent and further examinations are needed.

In the present study, the role of intraamygdaloid OTRs in social interaction was investigated in sham/neurotypical rats and in a VPA-induced autism model. We also studied the receptor-specificity of OT with OTR antagonist (ANT).

## 2. Materials and Methods

### 2.1. Subjects

Neurotypical and autistic signs displayed by male Wistar rats were examined. A VPA-induced autism model was employed. Female rats, being in the oestrus phase (determined from vaginal smears), were mated overnight. Dams received a single dose of VPA (Sigma-Aldrich Kft., Budapest, Hungary, P4543; 500 mg/bwkg ip.) dissolved in saline at a concentration of 250 mg/mL on the 12.5th day of the gestation [13]. Autistic signs were examined at the age of 4 weeks of the descendant animals with social interaction and open field test. Control females were injected with physiological saline. Dams were allowed to raise their own litters in their individual home cages.

In our experiments, 65 male (neurotypical and autistic signs showing) offspring Wistar rats were used in accordance with institutional (BA02/2000-8/2012 and BA02/2000-64/2017, University of Pécs Medical School approved by the National Scientific Ethical Community on Animal Experimentation of Hungary), national (Hungarian Government Decree, 40/2013 (II. 14.)), and international standards (European Community Council Directive, 86/609/EEC, 1986, 2010). All methods are reported in accordance with the ARRIVE guidelines. The rats were housed in a room where the temperature and lighting conditions were regulated (22 ± 2 °C; a 12:12 h cycle of light and darkness, with the lights switched on at 6:00 a.m.). They had unrestricted access to standard laboratory diet pellets (CRLT/N standard rodent feed pellet, Charles River Ltd., Budapest, Hungary) and tap water. All behavioral tests were conducted during the rats’ daylight phase, between 8:00 a.m. and 4:00 p.m.

### 2.2. Stereotaxic Surgery

Rats weighing 270–290 g underwent stereotaxic surgery to receive chronic, bilateral 22-gauge stainless steel guide cannulae. The surgical procedure was performed while the rats were under general anesthesia, which was maintained using intraperitoneal (ip) ketamine (80 mg/bwkg, (Calypsol Richter Gedeon) supplemented with diazepam (20 mg/bwkg, Seduxen Richter Gedeon). Cannulae were directed toward and 1 mm above the CeA (coordinates relative to bregma: AP: −2.3 mm, ML: ±4.1 mm, DV: −6.5 mm—as per the rat stereotaxic atlas [39]) during the surgery. The guide cannulae were secured in place using stainless steel screws and dental acrylic. Except during the drug administration, the guide cannulae were closed up with 27-gauge obturators. Rats received ip. antibiotic prophylaxis after the intervention. Prior to the experiments, animals had a week-long postoperative recovery, during which they were handled daily.

### 2.3. Drugs and Injection Procedure

Animals received bilateral microinjections 5 min prior to the test. During the microinjections, we paid attention to minimizing the effect impact on litter. Since littermates were more similar to one another than to animals from other litters [40], we divided them into different groups during the drug administration. A portion of the subjects was administered bilateral injections of 10 ng (9.93 pmol) of OT (Sigma-Aldrich Co., O6379) in a volume of 0.4 μL per side. The OT was dissolved in a sterile saline solution with a concentration of 0.15 M, along with 0.01 M Na-acetate and 0.01 M phosphate-buffered saline (PBS) at a pH of 7.4. For the control group, animals received this solution bilaterally as a vehicle in the same volume as that used for OT injections. In order to investigate receptor-specific effects, we employed the OT receptor antagonist L-2540 [Sigma-Aldrich Co., L-368-899, 20 ng (16.92 pmol)/0.4 μL]. The antagonist was diluted in a saline solution with a concentration of 0.15 M, containing 0.01 M Na-acetate and 0.01 M PBS at a pH of 7.4. Doses were defined based on our previous results and are reported as dose-per-side values [13,41].

Prior to application, the solutions were stored at a temperature of +4 °C. The drugs or the vehicles were microinjected bilaterally using a 30-gauge stainless steel injection tube, which extended 1 mm below the tips of the implanted guide cannulae. The injection cannula was connected to a 10 μL Hamilton microsyringe (Hamilton Co., Bonaduz, Switzerland) via polyethylene tubing (PE-10). All injections were administered using a syringe pump, delivering a volume of 0.4 μL (Cole Parmer, IITC, Life Sci. Instruments, Vernon Hills, IL, USA) over a 60-s period. Following the injections, the cannulae were left in place for an additional 60 s to allow for diffusion. The rats were manually restrained during the injection procedure. Animals were placed into the apparatus of social interaction tests after the microinjections.

Eight groups were involved in the experiment: (1) control group (neurotypical intact male Wistar rats receiving vehicle); (2) 10 ng OT group (neurotypical rats, receiving bilateral intraamygdaloid 10 ng oxytocin treatment); (3) ANT + OT (neurotypical rats, receiving 10 ng oxytocin after 20 ng oxytocin receptor antagonist pretreatment); (4) ANT (neurotypical rats, receiving 20 ng oxytocin receptor antagonist); (5) VPA (intrauterine valproate treated animals with autistic-like behavior, receiving vehicle); (6) VPA + 10 ng OT (intrauterine valproate treated animals with autistic-like behavior, receiving bilateral intraamygdaloid 10 ng oxytocin treatment); (7) VPA + ANT + OT (intrauterine valproate treated animals with autistic-like behavior, receiving 10 ng oxytocin after 20 ng oxytocin receptor antagonist pretreatment); (8) VPA + ANT (intrauterine valproate treated animals with autistic-like behavior, receiving 20 ng oxytocin receptor antagonist).

### 2.4. Social Interaction Test

**Figure 1:**
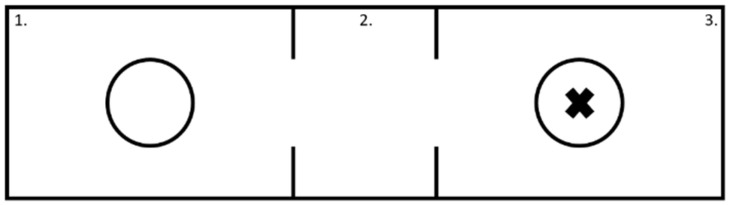
Schematic illustration of the three-chamber social interaction test. The apparatus consists of three parts: 1. non-social chamber; 2. central part; 3. social chamber. Circles represent wire cages; ✖ represents the unfamiliar rat in the cage.

Three-chamber social interaction test (also known as Crawley’s sociability and preference for social novelty test) is an effective way of studying rodents’ social behavior [42]. The apparatus (150 × 40 × 40 cm) was divided into three parts (shown in Figure 1), two chambers (60 × 40 cm) with circular wire cages and a central part (30 × 40 cm). One of the cages was empty (non-social zone), while an unfamiliar, neurotypical male rat with the same body weight as the examined animals was placed into the other cage (social zone). This rat was habituated for 5 min per day for 3 days before the experiments and had no previous interaction with the studied rats. After the microinjections, the examined animals were placed into the central part and spent 5 min in the setup, during which they could move freely in the apparatus. Time spent in the social and the non-social zone (data shown in Figure 3 and Figure 4) and the covered distance were measured. During the experiment, sociability was expressed as Sociability Index (SI), which was described as the ratio between the time spent in the social zone to the non-social zone [41,43] (data shown in Figure 5). The tests were performed in a climatized and sound-proof room. We recorded the behavior of the animals with the camera and used Noldus EthoVision Basic software (Noldus Information Technology B.V., Wageningen, The Netherlands) to analyze it. The arena was cleaned with Quatricide and dried with paper towels after each trial.

### 2.5. Histology

At the conclusion of the experiments, the rats were administered an overdose of Calypsol and Seduxen in a 4:1 ratio. They were then perfused transcardially with isotonic saline, followed by a 10% formalin solution. After one week of postfixation, their brains were frozen, sectioned into 40 μm thick serial sections, and stained with Cresyl-violet. The injection sites were reconstructed based on the rat brain’s stereotaxic atlas [39]. Only data from rats with accurately positioned cannulae were included in the subsequent analysis.

### 2.6. Statistical Analysis

Data are presented as mean ± standard error of the mean (S.E.M.). One-way and two-way ANOVA were employed, followed by Tukey’s post hoc analysis. Statistical significance was established at *p* < 0.05 (IBM SPSS Statistics 26).

## 3. Results

### 3.1. Histology

**Figure 2:**
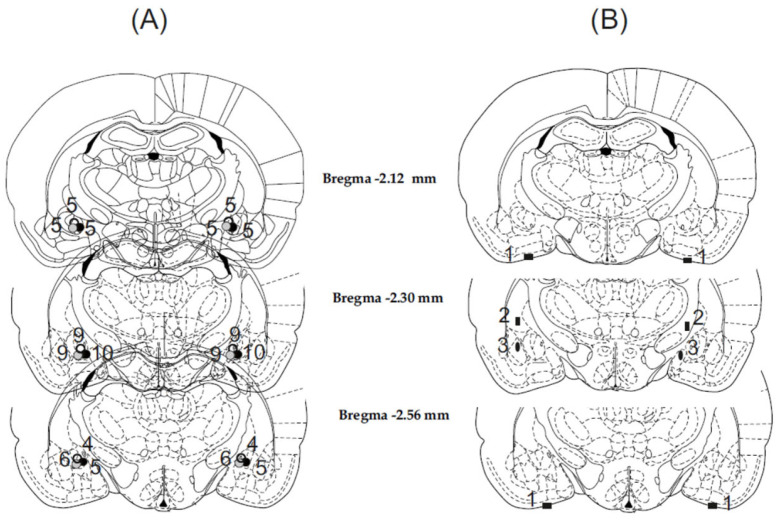
Displaying the reconstructed injection sites. In panel (**A**), correct bilateral injection placements are represented by circles in the CeA, with a total of 58 cases (*n* = 58). In panel (**B**), incorrect injection placements are indicated, comprising 7 cases *(n* = 7). The brain structure diagrams of the coronal sections are adapted from the Paxinos and Watson stereotaxic atlas [39]. The numbers on the diagrams indicate the anterior–posterior distances from the bregma in millimeters. In panel (**B**), identical symbols denote coherent injection sites of bilateral injections. The numbers displayed above the marked sites in both panels (**A**,**B**) represent the number of animals corresponding to each site.

Histological analysis revealed precise and symmetrical placement of the cannulae in the target area (CeA) in 58 out of 65 animals. The tracks created by the cannulae and the positions of the tips were determined based on the presence of debris and moderate glial proliferation. A schematic illustration depicting the locations of the cannula placements is shown in the accompanying Figure 2.

The 7 rats with misplaced injection sites were excluded from the subsequent analysis, as shown in Figure 2B. Among these rats, two cases involved cannula tips symmetrically entering the cerebrospinal fluid space at the base of the brain. In another two cases, the cannula tips were positioned laterally or medially 1 mm above the amygdala, resulting in injections being made in the caudate putamen on one side and the internal capsule on the other side. In the remaining three cases, the cannula tips were located laterally or medially to the target area, leading to the injections in the lateral and basolateral amygdala or in the medial nucleus of the amygdala. Due to these incorrect and varied placements, the behavioral data obtained from these rats were insufficient to draw far-reaching conclusions.

### 3.2. Social Interaction Test

**Figure 3:**
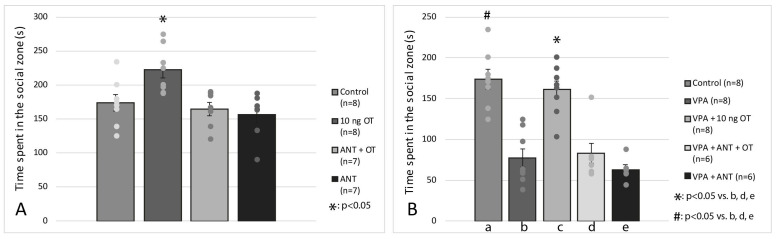
Effects of oxytocin (OT) and oxytocin receptor antagonist (ANT) on sham animals (**A**) and on rats showing autistic signs (**B**) in social interaction test. Columns represent mean time (±S.E.M.) spent in the social zone. Control: sham male Wistar rats receiving vehicle (*n* = 8); 10 ng. OT: sham rats receiving intraamygdaloid 10 ng oxytocin (*n* = 8). ANT + OT: sham rats treated with 20 ng oxytocin receptor antagonist before 10 ng oxytocin microinjection (*n* = 7). ANT: sham rats microinjected with 20 ng oxytocin receptor antagonist (*n* = 7). VPA: intrauterine valproate treated rats showing autistic-like behavior, receiving vehicle (*n* = 8). VPA + 10 ng OT: intrauterine valproate treated animals showing autistic-like behavior, receiving intraamygdaloid 10 ng oxytocin (*n* = 8). VPA + ANT + OT: intrauterine valproate treated animals with autistic-like behavior treated with 20 ng oxytocin receptor antagonist before 10 ng oxytocin microinjection (*n* = 6). VPA + ANT: intrauterine valproate treated animals with autistic-like behavior microinjected with 20 ng oxytocin receptor antagonist (*n* = 6); #, *: *p* < 0.05; for more explanation, see the text.

The effects of intraamygdaloid OT and ANT on time spent in the social zone of the apparatus are shown in Figure 3A. Based on one-way ANOVA analysis, there was a significant difference among the groups [F(3, 29) = 6.402, *p* < 0.01]. Tukey post hoc test revealed that the 10 ng OT-treated group (*n* = 8) spent significantly more time in the social zone than the control group (*n* = 8, *p* < 0.05) or ANT + OT-treated rats (*n* = 7, *p* < 0.05) or ANT-treated animals (*n* = 7, *p* < 0.05).

Figure 3B illustrates the effects of intrauterine VPA, intraamygdaloid OT, and ANT on time spent in the social zone of the apparatus. For analysis of social interaction, data of errors were processed with two-way ANOVA, with intrauterine treatment (VPA vs. Vehicle) and groups receiving different intraamygdaloid treatments (Vehicle, OT, ANT + OT, ANT). According to the results of a two-way ANOVA, there was a significant difference between intrauterine treatment (VPA vs. Vehicle) [F(1, 36) = 39.895, *p* < 0.05], and there was a significant difference between intraamygdaloid treatments (Vehicle, OT, ANT + OT, ANT) [F(3, 36) = 15.575, *p* < 0.05] along with a significant interaction between intrauterine treatment and intraamygdaloid treatments [F(3, 35) = 20.888, *p* < 0.05]. The Tukey post hoc test further showed that rats treated with VPA + 10 ng OT (*n* = 8) spent significantly more time in the social zone compared to the VPA group (*n* = 8, *p* < 0.05), VPA + ANT + OT (*n* = 6, *p* < 0.05) and VPA + ANT treated rats (*n* = 6, *p* < 0.05). There was no significant difference between the control animals (*n* = 8) and the VPA + 10 ng OT-treated rats (*n* = 8, N.S.). Pretreatment with the OT receptor antagonist (ANT) prevented the effect of 10 ng OT on time spent in the social zone. The ANT itself did not influence the time spent in the social zone in VPA-treated rats. No statistical difference was observed among the VPA, VPA + ANT + OT, and VPA + ANT-treated groups in terms of the time spent in the social zone. The Tukey post hoc test also revealed that control rats (*n* = 8) spent significantly more time in the social zone of the apparatus compared to the VPA group (*n* = 8, *p* < 0.05).

**Figure 4:**
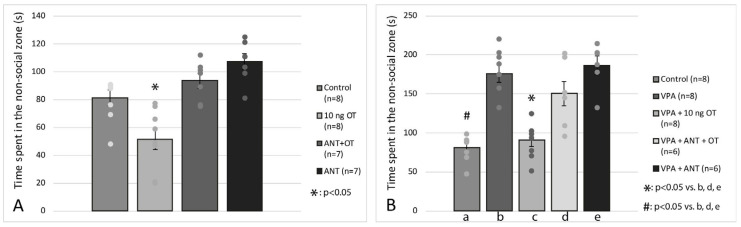
Time spent in the non-social zone is shown. (**A**) represents the effects of OT and/or ANT on sham rats and (**B**) on VPA-treated animals. #, *: *p* < 0.05; for more explanation, see the text.

The effects of intraamygdaloid OT and ANT on time spent in the non-social zone is shown in Figure 4 A. Based on one-way ANOVA analysis, there was a significant difference among the groups [F(3, 29) = 14.171, *p* < 0.001]. Tukey post hoc test revealed that the 10 ng OT-treated group (*n* = 8) spent significantly less time in the non-social zone of the apparatus than the control animals (*n* = 8, *p* < 0.05) or ANT + OT-treated rats (*n* = 7, *p* < 0.05) or ANT treated rats (*n* = 7, *p* < 0.05). Figure 4B shows the effects of OT and/or ANT microinjection on intrauterine VPA-treated rats. Two-way ANOVA analysis was applied, there was a significant difference between intrauterine treatment (VPA vs. Vehicle) [F(1, 36) = 41.876, *p* < 0.05] and there was a significant difference between intraamygdaloid treatments (Vehicle, OT, ANT + OT, ANT) [F(3, 36) = 16.103, *p* < 0.05] along with a significant interaction between intrauterine treatment and intraamygdaloid treatment [F(3, 35) = 20.229, *p* < 0.05]. Post hoc Tukey test revealed that rats treated with VPA + 10 ng OT (*n* = 8) spent significantly less time in the non-social zone compared to the VPA group (*n* = 8, *p* < 0.05), VPA + ANT + OT (*n* = 6, *p* < 0.05), and VPA + ANT treated rats (*n* = 6, *p* < 0.05). There was no significant difference between the control animals (*n* = 8) and the VPA + 10 ng OT-treated rats (*n* = 8, N.S.). No statistical difference was observed among the VPA, VPA + ANT + OT, and VPA + ANT treated groups in terms of the time spent in the non-social zone. The Tukey post hoc test also revealed that control rats (*n* = 8) spent significantly less time in the non-social zone of the apparatus compared to the VPA group (*n* = 8, *p* < 0.05).

**Figure 5:**
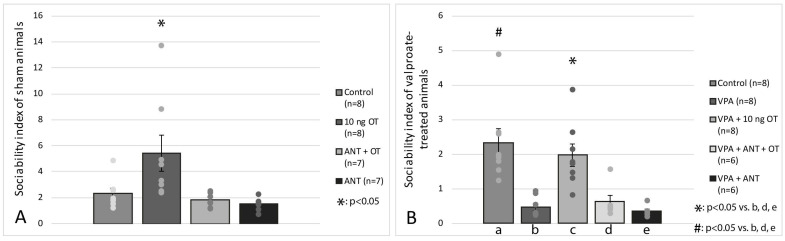
Shows the sociability index. (**A**) represents the effects of OT and/or ANT on sham rats and (**B**) on VP- treated animals. #, *: *p* < 0.05; for more explanation, see the text.

The effects of intraamygdaloid OT and ANT on SI are shown in Figure 5. Based on one-way ANOVA analysis, there was a significant difference among the groups [F(3, 29) = 5.00, *p* < 0.01]. Tukey post hoc test revealed that the 10 ng OT-treated animals (*n* = 8) had significantly higher SI than the control group (*n* = 8, *p* < 0.05) or ANT + OT-treated animals (*n* = 7, *p* < 0.05) or ANT-treated rats (*n* = 7, *p* < 0.05). Figure 5B shows the effects of OT and/or ANT microinjection on intrauterine VPA-treated rats. Two-way ANOVA analysis was applied; there was a significant difference between intrauterine treatment (VPA vs. Vehicle) [F(1, 36) = 25.175, *p* < 0.05], and there was a significant difference between intraamygdaloid treatments (Vehicle, OT, ANT + OT, ANT) [F(3, 36) = 7.817, *p* < 0.05] along with a significant interaction between intrauterine treatment and intraamygdaloid treatment [F(3, 35) = 11.586, *p* < 0.05]. Post hoc Tukey test revealed that rats treated with VPA + 10 ng OT (*n* = 8) had significantly higher SI compared to the VPA group (*n* = 8, *p* < 0.05), VPA + ANT + OT (*n* = 6, *p* < 0.05), and VPA + ANT treated rats (*n* = 6, *p* < 0.05). There was no significant difference between the control animals (*n* = 8) and the VPA + 10 ng OT-treated rats (*n* = 8, N.S.). The post hoc Tukey test also revealed that control rats (*n* = 8) showed significantly higher SI compared to the VPA group (*n* = 8, *p* < 0.05).

The locomotor activity of the animals (covered distance) was also measured, but no significant difference was found among the groups (data shown in Table 1).

## 4. Discussion

About 1.5% of children are affected by ASD worldwide, and pharmacological treatment is still not found [6]. Non-pharmacological therapies (such as music therapy, cognitive-behavioral therapy, social-behavioral therapy, ketogenic diet) play an important role in the treatment of ASD, and some pharmacological interventions are known as well (e.g., SSRIs, alpha-2 adrenergic receptor agonists, antipsychotic drugs) but all of these provide only symptomatic treatment and could have several and severe side effects [6,44]. The VPA-induced autism model provides an opportunity to investigate the pathophysiology underlying autistic behavior and could bring us closer to the proper medical treatment of ASD. Schneider et al., were the first to evaluate behavior in detail in the VPA model [45]. They found a decreased number of and increased latency to social behaviors in rats who received a single dose of intrauterine VPA on day 12.5 of the gestation [45]. Social-behavioral abnormalities have also been correlated with chronic depletion of the gut microbiota in rats, which makes the pathophysiology even more complex and more difficult to understand [46]. Social impairment was detectable in our experiments in intrauterine VPA-treated rats as well. Our aim was to study the effect of intraamygdaloid OT treatment on social interaction in neurotypical rats and in the VPA-induced autism model.

OT is well-known for its effect on social and parental behaviors, partner selection, and developing eye contact [30,47]. Changes in the oxytocinergic system and/or reduction in endogenous OT levels are in close connection with the impairment of social interaction and communication, which is a core symptom of ASD [47]. Based on previous results, OT could be a potential therapy for ASD. A single-dose intranasal OT treatment has improved impaired social interaction for 2 h in a VPA-induced mouse model, and the effect lasted for 24 h when it was administered for 2 weeks [48,49]. Clinical trials have also demonstrated a positive effect of intranasal OT treatment in patients with ASD; however, the exact dose getting through the blood–brain barrier and long-term effects are still to be examined, and the results are sometimes contradictory [50,51]. Not only the level of OT but also the expression and/or the morphology of OT receptors could be different in ASD. OT receptors can be found in a large number in the amygdala [27], which is part of the social brain that is impaired in ASD, but the effect of OT microinjected into the CeA on social interaction in the autism animal model has not been examined yet. Bertelsen et al., have found a reduction in OT receptor binding in the amygdala in the VPA autism model [48]. Our aim was to investigate the role of intraamygdaloid OT receptors in social interaction in sham and in autistic signs showing male rats. During the microinjections, we paid attention to minimizing the impact of the litter effect. Since littermates are more similar to one another than to animals from other litters [40], we divided them into different groups during the drug administration.

Three-chamber social interaction test has been previously used in the VPA model; therefore, our data can be compared with previous results [12,52]. This apparatus is proper to examine social interaction since by placing the unfamiliar animal into a wire cage, nose contact is allowed, but we can prevent direct physical contact, aggression, and potential injury; moreover, behavior influenced by dominance can also be excluded [42].

Due to the 10 ng intraamygdaloid OT treatment, we found a significant improvement in impaired social interaction in the VPA-induced autism model. We have not found significant differences among the groups in time spent in the non-social zone or in the covered distance (neither the OT nor the ANT treatment had a major effect on it). Consequently, we could establish that the increase in time spent on social interaction was presumably the result of intraamygdaloid OT microinjection and not an unspecific effect on the general activity of the animals.

Impairment of social interaction and anxiety are closely linked, and changes in the oxytocinergic system and/or the endogenous level of OT could be the common cause [35]. Lebowitz et al., have found significantly lower salivary levels of OT in youths with a separation anxiety disorder than anxious youths without separation anxiety disorder [53]. Low postpartum plasma OT levels have been linked to separation anxiety during pregnancy and to poor mother–infant bonding [41,53]. Most patients with ASD suffer from anxiety to different degrees as well [6]. In our previous study, intraamygdaloid OT treatment had a significant anxiolytic effect on intrauterine VPA-treated rats in elevated plus maze test [41].

We also examined the receptor-specificity of OT with ANT (L-2540), which is a non-peptide substance blocking the OT receptors selectively. Peptide antagonists exist as well, but receptor-specificity could not be studied precisely since they could act as partial agonists [54]. Given alone, the ANT did not affect time spent on social interaction significantly, neither in sham nor in autistic-signs-showing rats. ANT pretreatment has prevented the effect of OT, which suggests that the effect of OT was receptor specific. Based on our results, intraamygdaloid OT receptors play a key role in developing social interactions. In case the ANT had not prevented the effects of OT, the possibility of activating other signal pathways by binding to vasopressin V1a and/or V1b receptors should be taken into consideration. These two neuropeptides have a very similar structure, and their receptors belong to the same receptor superfamily; therefore, they could bind to each other’s receptor [55]. This could contribute to the bell-shaped dose-dependent effect of OT [56]. Another possible theory could be the activation of the mesocorticolimbic dopaminergic system since it has more connections with oxytocinergic pathways. Oxytocinergic neurons in the preoptic, supraoptic, and paraventricular nuclei of the hypothalamus have D2, D3, and D4 dopamine receptors [57]. Moreover, dopamine can regulate the expression of intraamygdaloid OT receptors via the protein kinase A pathway [28]. Based on our previous results, we can establish that the two systems can modulate each other’s effects since dopamine D2 receptor antagonist pretreatment has blocked the anxiolytic effect of OT in elevated plus maze test [58].

OT receptor is modulated by gonadal hormones, and this could lead to gender differences in the morphology of the receptor [27]. Sex hormones can also affect the endogenous levels of OT and vasopressin [55]. Changes in the vasopressin system could be an underlying cause of the male bias of ASD—it tends to be fourfold more frequent in males than in females [6,55]. During our experiments, only male rats were examined; therefore, our results do not provide information on gender differences. Another possible limitation of our studies is that there was no significant difference in the age of our animals since the stereotaxic atlas [39] we used to define the correct coordinates during the cannula implantation is designed for rats weighing about 270–290 g. Furthermore, only short-term effects of a single dose of OT were examined since volume loading is limited due to the possible damage to brain structures, although observation of long-term effects would be advantageous, too.

All in all, the pathophysiology of ASD is quite complex and could be regulated or modified by multiple factors, which makes it hard to understand; therefore, medical treatment is still a difficulty health care must face. Our data may provide relevant information for clinical research projects and for drug development.

## 5. Conclusions

Our results indicate that bilateral 10 ng OT microinjection into the CeA increases the time spent on social interaction and sociability index. These effects have been observed in rats showing autistic-like behavior and in neurotypical rats as well. We have also revealed that the effects of intraamygdaloid OT on social interaction are OT receptor specific.

## Figures and Tables

**Table 1:** Covered distance during the social interaction test (cm); data represent mean ± S.E.M. Control: sham male Wistar rats, receiving vehicle (*n* = 8); 10 ng OT: sham rats, receiving intraamygdaloid 10 ng oxytocin (*n* = 8); ANT + OT: sham rats treated with 20 ng oxytocin receptor antagonist before 10 ng OT microinjection (*n* = 7); ANT: sham rats microinjected with 20 ng oxytocin receptor antagonist (*n* = 7); VPA: intrauterine valproate-treated rats showing autistic-like behavior, receiving vehicle (*n* = 8); VPA + 10 ng OT: intrauterine valproate-treated animals showing autistic-like behavior, receiving intraamygdaloid 10 ng oxytocin (*n* = 8); VPA + ANT + OT: intrauterine valproate-treated animals with autistic-like behavior, treated with 20 ng oxytocin receptor antagonist before 10 ng oxytocin microinjection (*n* = 6); VPA + ANT: intrauterine valproate-treated animals with autistic-like behavior, microinjected with 20 ng oxytocin receptor antagonist (*n* = 6).

	Covered Distance (cm)
Control	1717.33 ± 55.33
10 ng OT	1821.25 ± 61.86
ANT + OT	1788.50 ± 48.45
ANT	1675.52 ± 90.12
VPA	1699.52 ± 52.66
VPA + 10 ng OT	1709.33 ± 67.71
VPA + ANT + OT	1785.15 ± 68.89
VPA + ANT	1689.58 ± 98.88

## Data Availability

The data supporting the reported results of this study are available here: https://drive.google.com/drive/folders/1ZgRKeetDs4UZoV7bXQnptjen9FILPyfq?usp=sharing (First access 20 November 2022, 9:47:45 AM).
